# Mixed Tree Nuts, Cognition, and Gut Microbiota: A 4-Week, Placebo-Controlled, Randomized Crossover Trial in Healthy Nonelderly Adults

**DOI:** 10.1093/jn/nxac228

**Published:** 2022-10-06

**Authors:** Crystal F Haskell-Ramsay, Fiona L Dodd, Darren Smith, Lewis Cuthbertson, Andrew Nelson, John K Lodge, Philippa A Jackson

**Affiliations:** Department of Psychology, Northumbria University, Newcastle upon Tyne, United Kingdom; Brain, Performance and Nutrition Research Centre, Northumbria University, Newcastle upon Tyne, United Kingdom; Department of Applied Sciences, Northumbria University, Newcastle upon Tyne, United Kingdom; Department of Applied Sciences, Northumbria University, Newcastle upon Tyne, United Kingdom; Department of Applied Sciences, Northumbria University, Newcastle upon Tyne, United Kingdom; School of Human Sciences, London Metropolitan University, London, United Kingdom; Brain, Performance and Nutrition Research Centre, Northumbria University, Newcastle upon Tyne, United Kingdom

**Keywords:** cognitive function, cognition, mood, gut microbiota, metabolomics, nuts, healthy adults

## Abstract

**Background:**

Beneficial effects of nut supplementation on cognitive function have previously been demonstrated in young and older adults. Alterations to gut microbiota have also been shown following tree nut consumption. However, no data exists on the effects of nuts on cognition and intestinal microbial communities assessed within the same study.

**Objectives:**

The study aimed to examine the effects of daily consumption of tree nuts for 4 wk on cognitive function (primary outcome), mood, metabolomics, and gut microbial species (secondary outcomes) in healthy, nonelderly adults.

**Methods:**

This randomized, placebo-controlled, double-blind, counterbalanced crossover study assessed the effects of 4 wk of supplementation with 30 g/d mixed tree nuts versus placebo on cognition and mood in 79 healthy adults aged 18–49 y. Metabolic responses, gut bacterial community structure, and the potential for these to impact cognition were explored using a multi-omic approach. Bacterial community analysis was conducted in Quantitative Insights Into Microbial Ecology 2 (QIIME2).

**Results:**

Mixed model analysis indicated that nut consumption led to significant improvements to accuracy (placebo *M* = 92.2% compared with NUTS *M* = 94.5%; *P* = 0.019) and speed of response (placebo *M* = 788 ms compared with NUTS *M* = 757 ms; *P* = 0.004) on a picture recognition task. No significant changes to bacterial community α or β diversity were observed when comparing nut consumption to the placebo arm. However, an unclassified *Lachnospiraceae* amplicon sequence variant (ASV) was significantly enriched in participants when supplemented with nuts (*P* = 0.015). No correlations were observed between the changes to picture recognition and the changes to the unclassified *Lachnospiraceae* ASV. There were no significant changes to the urinary metabolome.

**Conclusions:**

These findings indicate a positive effect of nut on cognition following only 4 wk of consumption in a healthy nonelderly sample, as well as upregulation of a microbial taxa associated with gut health. The effects appear to be independent of one another, but further exploration is required in those experiencing cognitive decline and/or gut dysbiosis.

## Introduction

Cognitive impairment is a growing worldwide health concern as our population ages. In the absence of effective pharmaceutical treatments, modifiable lifestyle factors such as nutrition represent crucial targets in preventing cognitive decline. The study of whole foods, such as nuts, is particularly valuable as they provide a range of nutrients, including unsaturated FAs, dietary fiber, vitamins, minerals, L-arginine, and phenolic compounds; and represent an easily adopted dietary change for consumers. Nuts beneficially modulate a broad spectrum of parameters including cardiometabolic function (1, 2), lipid profile (3), and weight gain (4); all of which have the potential to impact cognition.

The consumption of nuts has been linked to better cognition in those aged 20–90 y (5–7). However, long-term (6-y) intake was not associated with cognitive decline in women aged ≥70 y (7) and an association to 5-y cognitive decline in men and women aged >45 y was lost following adjustment for cardiovascular risk factors (6). This indicates a role for cardiovascular effects, which is supported by cognitive improvements shown following Mediterranean diet plus mixed tree nuts in those at high cardiovascular risk (8). A specific benefit for walnuts has been indicated in epidemiological studies (5, 9). In intervention trials, a 2-y multi-center study showed cohort-specific cognitive improvements in cognitively healthy elders, potentially related to lower education status and suboptimal nutrition in the cohort affected (10). An 8-wk walnut supplementation in adults aged 18–25 y also showed a specific improvement to inferential verbal reasoning (11).

Alterations to gut microbiota have been highlighted as a potential mechanism for diet-induced changes to cognition (12). Studies of the gut microbiome have shown modulation of *Ruminococcaceae, Bifidobacteria*, and *Clostridium* clusters XIVa species following walnut (13, 14). Whilst almond consumption was shown to increase α diversity (15) and relative abundances of *Lachnospira, Roseburia*, and *Dialister* (16). Previous metabolomic research has highlighted biomarkers of nut exposure and shown these to be inversely correlated with severity of metabolic syndrome (17) and cognitive decline (18).

The lack of association between nut consumption and 5–6-y cognitive decline (6, 7) suggests that early nutritional intervention may be needed to ensure a positive impact on cognitive decline in older age. This study investigated the cognitive and mood effects of mixed tree nut supplementation in adults aged 18–49 y. The impact of nut consumption on metabolic responses and gut microbiota colonization and the potential for these to impact cognition were explored using a multi-omic approach to ascertain their role in any effects on cognition. This represents the first intervention study of a whole food incorporating cognitive, metabolomic, and gut microbiota responses.

## Methods

### Design

A randomized, placebo-controlled, double-blind, counterbalanced crossover design was utilized. Participants were assessed immediately prior to and following 2 4-wk crossover treatment periods. The study was approved by the University Ethical Approval System at Northumbria University and was conducted according to the Declaration of Helsinki (2013). The study was registered at clinicaltrials.gov as NCT03500601.

### Participants

Volunteers were recruited through opportunity sampling within Newcastle upon-Tyne, United Kingdom, and the surrounding areas. A power calculation based upon previous repeated measures data showing an effect size of f = 0.2 (11) indicated that 76 participants with a complete data set would allow the detection of significant effects with a power of 0.8 at α = 0.05. To allow for drop-out, 81 males and females (aged 18–49 y) were randomly assigned to treatment. All participants were healthy, nonsmokers, and had a BMI within the range of 18.5–30 kg/m^2^. Participants confirmed that they did not have any pre-existing medical conditions, were not habitually taking any medication (excluding the contraceptive pill), had not habitually used supplements within the last month, and had not taken antibiotics in the 6 wk prior to visit 1. In order to avoid potential issues with nut allergy or intolerance, all participants self-reported that they consumed nuts but not more than twice per week. Participants were reimbursed £165 for their time.

### Treatment

Participants received 28 d of 30 g mixed tree nuts (15 g walnuts, 7.5 g almonds, 7.5 g hazelnuts) and 28 d of placebo (microcrystalline cellulose) with 4-wk wash out between treatments. Treatment order was determined using computer-generated random allocation conducted by an independent 3rd party. In order to blind participants to the treatment received, they were informed that the aim of the study was to explore effects of “components of nuts” and thus placebo capsules were provided alongside nuts to facilitate this deception. Treatments were provided as: Active treatment (NUTS), whereby participants were instructed to consume 1 × 30 g bag of nuts and 1 (placebo) capsule per day; and placebo (PLA), whereby participants were instructed to consume 2 (placebo) capsules per day. Participants were directed to take their treatment daily for a period of 28 d. Success of blinding was assessed via treatment guess at the end of the final visit. Compliance was assessed via treatment diaries, weighing of returned nuts, and pill counting and was conducted by a researcher who was not otherwise involved with the study. This researcher also distributed and received returned treatments in order to maintain blinding of researchers.

### Subjective measures

#### Profile of mood states

Participants were required to complete the paper and pencil version of profile of mood states (POMS 2), which provides scales of tension-anxiety, depression-dejection, anger-hostility, vigor-activity, fatigue-inertia, confusion-bewilderment, and friendliness. A score was obtained for each scale and “total mood disturbance” was computed from tension, depression, anger, vigor, fatigue, and confusion responses (19).

#### Bond–Lader visual analogue scales

Participants were presented with 16-line scales anchored at either side by an adjective describing a mood. Participants clicked at a point on each scale to represent how they were feeling at that point in time. Each scale was scored out of 100 and from these scales 3 composite scores were calculated describing feelings of “Alert”, “Calm”, and “Content”, also presented as a score out of 100 (20).

### Cognitive measures

All cognitive tasks were conducted via laptop computer using the Computerized Mental Performance Assessment System (COMPASS, Northumbria University, Newcastle upon Tyne, United Kingdom). COMPASS is a software platform for the presentation of classic and bespoke computerized cognitive tasks, which has previously shown sensitivity in detecting cognitive effects of nutritional interventions (21–23). Given the paucity of evidence regarding the effects of nut consumption on cognitive performance outcomes, tasks were chosen to cover a range of cognitive domains including memory, attention, and executive function. All stimuli were randomized across participants and assessments, and on-screen instructions provided prior to each task allowed the participant to control their own progress throughout. Tasks were presented in the same order on each occasion and, except for the paper and pencil tasks (immediate and delayed word recall), responses were made using a response pad or mouse (computerized location learning task; peg and ball). See **Table 1** for task descriptors and order.

**TABLE 1:** Cognitive task descriptors, scoring, and domain in order of completion

Task	Descriptor	Scoring	Domain
Word presentation	A list of words is displayed on the screen, 1 word at a time. In this case, 15 words were presented with a display time of 1 s and interstimulus interval of 1 s	—	
Immediate word recall	Participants are instructed to write down the words that were presented. In this case, 60 s were given to complete the task	Number correct, errors	Episodic memory
Picture presentation	A series of photographic images are displayed on the screen, 1 at a time. In this case, 15 images were presented with a display time of 2 s and an interstimulus interval of 1 s	—	
Computerized location learning, learning phase	A grid containing pictures of objects is shown. In this case, the presentation duration was 15 s followed by an interval of 10 s. An empty grid is then shown, and participants are asked to relocate the objects to the correct location shown to them previously, with no time limit for responding. This is repeated 5 times during the learning phase, with a pause of 5 s between each trial	Total displacement score (sum of the errors for each object from each trial) and learning index (average relative difference in performance between trials)	Spatial memory
Choice reaction time	Arrows pointing left and right appear on the screen at irregular intervals. The participant is required to indicate the direction of the arrow as quickly as possible whenever an arrow is displayed, by pressing the corresponding button. In this case, 50 stimuli were presented	Accuracy (%) and reaction time for the correct responses (ms)	Attention
Rapid visual information processing	A series of digits appears on screen 1 at a time in a pseudorandom order and at the rate of 100 per min. Participants are required to make a response when they see 3 consecutive odd or even numbers. In this case the task lasted for 5 min	Accuracy (%), reaction time for the correct responses (ms), and false alarms	Attention
Numeric working memory	A series of numbers are displayed on the screen, 1 at a time. Participants are required to memorize these numbers as they appear. Once the series is complete, numbers are displayed 1 at a time and participants are required to indicate if each number was presented in the previous list or not. In this case, 3 trials were completed with 5 target numbers in each trial	Accuracy (%) and reaction time for the correct responses (ms)	Executive function
Logical reasoning	Statements pertaining to a pair of letters are presented on screen, these are followed by the pair of letters referred to. Participants are required to indicate whether each sentence correctly describes the pair of letters that follow it by selecting true or false. In this case 10 true and 10 false statements were presented	Accuracy (%) and reaction time for the correct responses (ms)	Executive function
Stroop	Words describing one of 4 colors (“RED”, “YELLOW”, ‘GREEN”, “BLUE”) are presented in different colored fonts on screen. In this case 60 “congruent” (word and font are the same color) or “incongruent” (word and font are different colors) were presented in a random order	Accuracy (%) and reaction time for the correct responses (ms)	Executive function
Peg and ball	Two configurations are shown on the screen each containing 3 colored balls on 1 of 3 pegs. Participants must arrange the balls on the starting configuration to match the position of balls in the goal configuration in the least number of moves possible	Average thinking time (ms), average completion time (ms), and errors	Executive function
Delayed word recall	Participants are instructed to write down the words that were presented to them at the beginning of the assessment. In this case, 60 s were given to complete the task	Number correct, errors	Episodic memory
Delayed picture recognition	All target pictures shown during picture presentation plus an equal number of decoys are displayed on the screen 1 at a time. Participants indicate if they remember seeing the picture earlier or not	Accuracy (%) and reaction time for the correct responses (ms)	Episodic memory
Delayed word recognition	All target words that were shown during word presentation plus an equal number of decoys are displayed on the screen 1 at a time. Participants indicate if they remember seeing the word earlier or not	Accuracy (%) and reaction time for the correct responses (ms)	Episodic memory
Computerized location learning recall	Participants are again asked to place the objects in the correct location on the empty grid with no further prompting	Delayed recall (final learning trial displacement score minus delayed displacement score)	Spatial memory

### Urine sampling

Urine samples were collected by the participant directly into 30 mL aseptic containers (Alpha Laboratories Ltd). Samples provided were the first urination of the day after the morning void. Upon receipt from the participant, samples were refrigerated (within 30 min) then (within ∼1 h of refrigeration), were poured into 3 × labeled, small screw cap tubes (Greiner Bio One Ltd) before being stored at –20°C prior to analysis.

### Stool sampling

Stool samples were collected by the participant at home on the morning (or within 24 h) of their testing visit using Fe-Col® fecal sample collection kits (Alpha laboratories Ltd). Participants were instructed to store their sample in the fridge (or a cool place) following collection. Upon receipt at the lab, samples were refrigerated, then (within ∼1 h) the collection tube was sanitized (Clinell wipes, GAMA Healthcare Ltd) before being stored at –80°C prior to analysis.

### Urinary metabolomic methods

Hydrophilic interaction LC-based analysis of urine samples was conducted on a subset of 40 participants using ultrahigh resolution LC and MS (24). Urine samples were normalized using the refractive index and prepared according to previous methods (24). Metabolite profiles of urine samples were generated on a Dionex 3000 Ultra HPLC system hyphenated to the Q-Exactive classic high-resolution MS system (ThermoScientific). All solvents and ionization agents used were of analytical grade or higher unless stated. The chromatographic separation was performed on a Water Acuity Ethylene Bridge Hybrid Amide analytical column (2.1 × 150 mm) with particle size of 1.7 ¼ micronat a flow rate of 400 ¼L/min, the column temperature was set to 45°C. The binary buffer system was as follows: Buffer A was MilliQ water and Buffer B was acetonitrile, both with 10 mM ammonium formate adjusted to pH 3.5 using formic acid.

The LC profile was as follows: T: 0 min 90% (B), T: 2 min 60% (B), T: 5 min 40% (B), T: 7.5 min 40% (B), T: 7.6 min 90% (B), T: 10 min 90% (B). A 3 ¼L injection was applied. The heated spray ionization source (HESI) was set to the following parameters: Sheath gas flow rate of 50, the aux gas flow rate was set to 13, and the sweep gas flow rate was 3. The spray voltage was set to 3.5 akV with a capillary temperature of 275°C. The Aux gas heater temperature was adjusted to 425°C. The mass (MS1) acquisition range was as follows: 75–1000 m/z units at a mass resolution of 35,000 at ∼7.6 scans per second, microscan: 1, lock mass: off. The automatic gain control (AGC) was set to 1e6 and the ion injection time was 100 mS^−1^. The data was acquired on both Positive and Negative mode polarity (independently), the setting for the negative mode was the same as positive ion mode except the voltage was set to 2.5 kV. The system was primed with a minimum of 10 sequential injections of pooled quality control (QC) to stabilize the HESI source and to check for chromatographic stability before initialing the batch analysis. Peak table generation and alignment were performed using compound discoverer 2.1 (ThermoScientific) with an alignment window of 0.25 min, mass tolerance of 5 ppm, and a signal intensity threshold of 200,000 counts with a signal to noise ratio of 5:1.

### Characterization and identification of discriminating features

Peak intensity tables from Compound Discoverer were processed using MetaboAnalyst© software (25). The full data set was autoscaled and log transformed. MetaboAnalyst© performed detailed multivariate and univariate analysis including Principal Component Analysis (26) that was used for data quality control and Partial Least Squares Discriminant Analysis (PLSDA) that was used to test for discrimination between sample groups. Cross validation tests were used to test the robustness of the model, using Q2 and R2 and classification metrics, and Variable Importance in Projection (VIP) data was used to rank the most discriminatory species. Annotation of identified metabolites was carried out according to level 2 of identification proposed by the Metabolomics Standards Initiative (27). The top discriminating features (VIP >2.5) in each mode underwent putative identification using established databases METLIN (28) and the Human Metabolome Database (29), but as no significant changes to the metabolome were observed, further identification of discriminatory metabolites was not performed.

### Bacterial community sequencing

DNA extraction was performed on 200 mg of stool sample using the DNeasy PowerSoil HTP 96 kit (QIAGEN) as per the manufacturers’ instructions. The purity of the genomic DNA was checked using the Nanodrop 1000 UV-Vis Spectrophotometer (ThermoScientific) with a A260:280 nm of >1.8 required to proceed to sequencing. Amplicon sequencing of the V4 region of the 16S rRNA gene was performed at NU-OMICs DNA sequencing research facility (Northumbria University) using the method described by (30) on an Illumina MiSeq Benchtop sequencer with V2 500 cycle chemistry. Data was uploaded to Sequence Read Archive (SRA) under BioProject ID PRJNA881306.

### Bacterial community analysis

The QIIME2 bioinformatics pipeline (31) was utilized to transform raw fastq files to amplicon sequence variants (ASVs) as described previously (32). Briefly, ASVs were generated using the DADA2 algorithm and sequences were classified using Greengenes database (v.13.8).

Data analysis and visualizations were carried out in R studio (Version R 3.6.1), using the phyloseq, vegan, ggplot2, gridextra, and scales packages. The α diversity metrics were calculated and significance of multiple continuous variables determined using the Pairwise Wilcoxon test with Bonferroni adjustment. The Wilcoxon test with False Discovery Rate (FDR) algorithm was used when comparing categorical variables. The β diversity was assessed using weighted Bray–Curtis dissimilarities and displayed using PCoA analysis and PERMANOVA was used to determine the significance of dissimilarity between groups.

### Procedure

Participants were required to attend the Brain, Performance and Nutrition Research Centre (Northumbria University, United Kingdom) on 5 separate visits. Screening visits took place a minimum of 1 wk prior to the first testing visit. Following informed consent, participants were screened for eligibility, provided demographic data, and had their blood pressure taken. Training on the cognitive tasks involved completing the tasks 5 times to eradicate practice effects. Participants were also given an FFQ (33) to complete at screening. Participants were instructed to refrain from consuming all nuts and nut products (other than those provided to them) from this point until the end of data collection and this was assessed via questionnaire at visits 1–4. They were then provided with the kit and instructions for collecting stool samples for visit 1.

**FIGURE 1:**
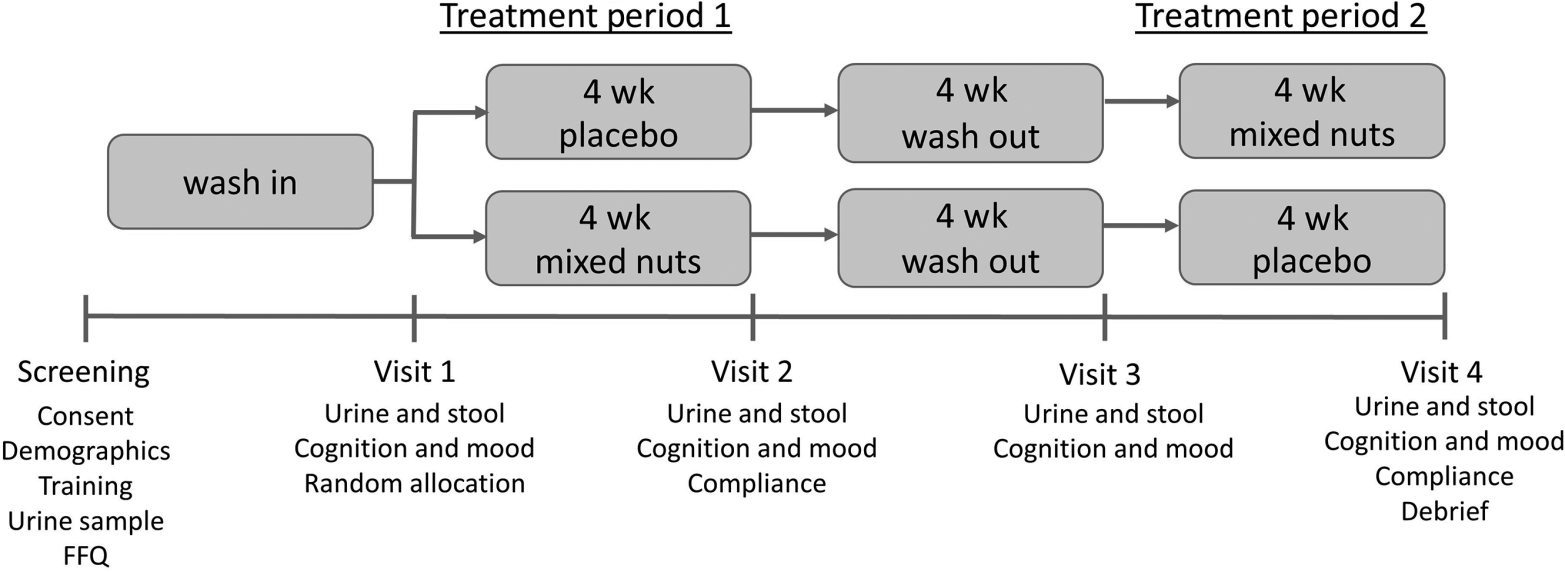
Schematic of study procedures.

Visits 1–4: Participants refrained from caffeine for a minimum of 10 h, the consumption of alcohol and intake of over-the-counter medication for 24 h, and systemic antihistamines for 48 h prior to each visit. Participants attended the laboratory at an agreed time in the morning following a light breakfast of cereal and/or toast at home 1 h (minimum) before the session commenced, with breakfast standardized across each visit. Prior to test procedures, eligibility criteria and concomitant medications were reviewed, and supplementation and adherence to nut intake restrictions confirmed. A urine sample was then provided, and stool sample collected prior to completion of the mood and cognitive measures (see **Figure 1**). Physical activity was assessed using the International Physical Activity Questionnaire (IPAQ; 34). Weight was also measured at each visit to assess any weight change due to supplementing with nuts. At visit 1, participants were randomly allocated to treatment order and at the end of visits 1 and 3 they were provided with treatment and instructed to take their first day's treatment that day once they had left the laboratory. They were also provided with a diary to complete date and time of taking their treatment each day. Compliance to treatment was assessed at visits 2 and 4 via diary and return of unused treatment. At visits 1–3 participants were provided with the kits and instructions to obtain their stool samples, so they could be returned at their next study visit. At the end of visit 4 participants were fully debriefed.

### Statistical analysis

The postdose cognitive and mood outcome measures, as well as microbiota identified as changing significantly, were modeled using the MIXED procedure in SPSS (version 26.0, IBM Corp.). This included the respective baseline values and the terms Treatment and Visit as fixed factors and Participant as a random factor. Significant interaction effects were followed up with Bonferroni corrected pairwise comparisons exploring the differential effects of treatment across visits and differential effects across visits within treatments. Further exploratory analysis was conducted on recognition measures to ascertain whether effects were specific to novel or original stimuli. Partial bivariate Pearson correlations were conducted on change from baseline values to assess any relation between significant changes in the gut microbiota and cognition following nut consumption adjusting for the potential confounding effects of age, sex, years in education, and BMI.

## Results

### Demographics

**FIGURE 2:**
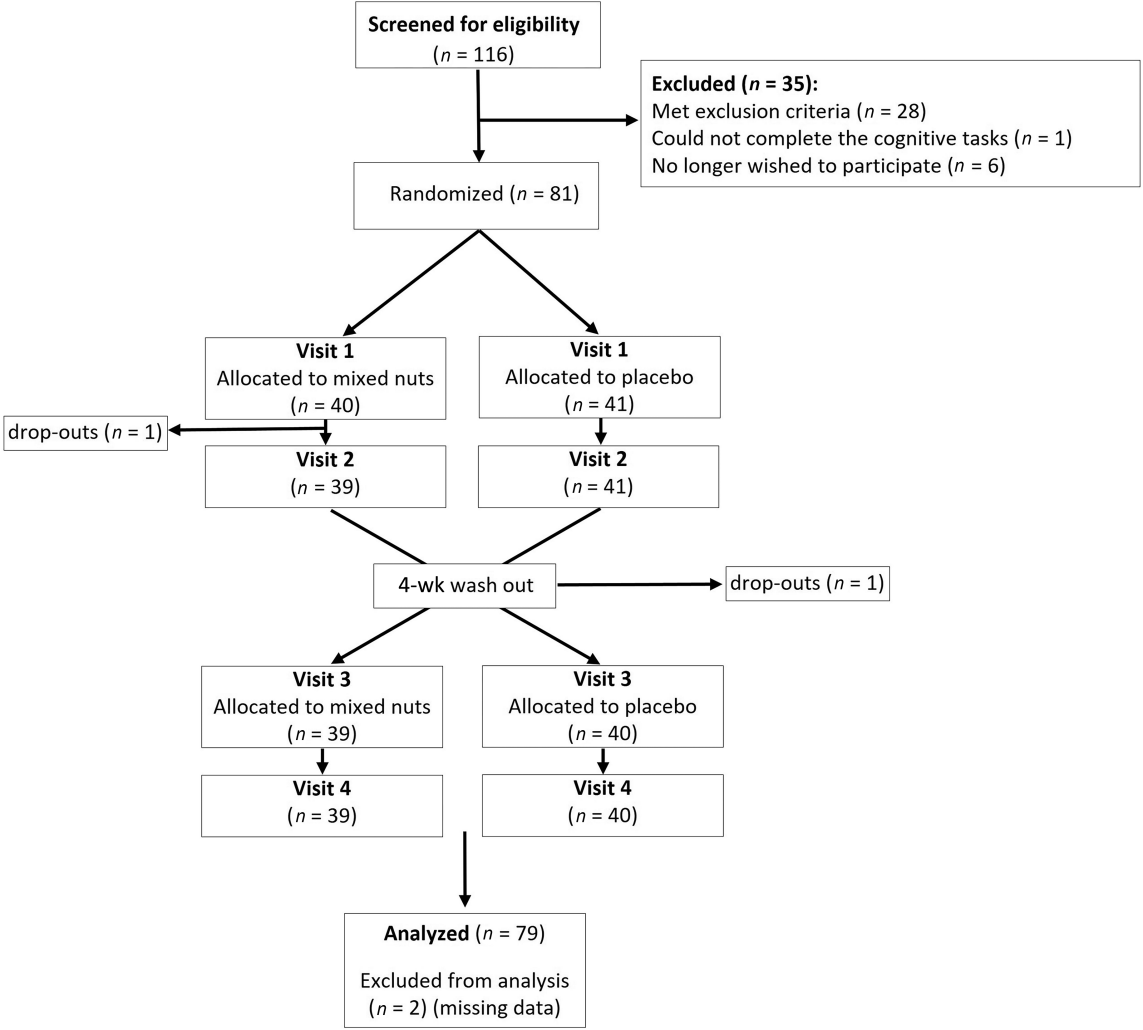
Participant disposition flow chart.

Seventy-nine participants (28 male) mean age: 29.9 y (SD: 8.12); BMI: 23.0 (SD 2.82), who consumed 4.02 (SD: 2.24) portions of nuts per month, completed the study (see **Figure 2**). Participants had an average of 17.6 y (SD: 3.16) in education and consumed 3.70 (SD: 1.81) portions of fruit and vegetables per day. Average systolic blood pressure was 117.9 (SD: 10.4); diastolic blood pressure 75.9 (SD: 7.03); and heart rate 71.9 (SD: 12.4). Eleven participants were vegetarian and 4 reported consuming food with added live cultures as part of their normal diet.

### Cognitive performance

#### Picture recognition accuracy

**FIGURE 3:**
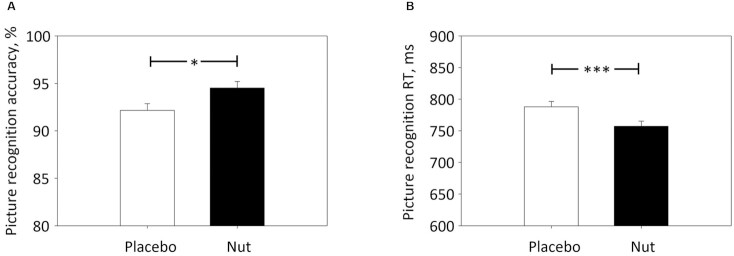
Estimated marginal means + SE derived from linear mixed model analysis for picture recognition accuracy (A) and picture recognition reaction time (RT) (B) in nonelderly adults who consumed mixed tree nuts and placebo, each for 4 wk. Significant treatment effect of nuts versus placebo *P <0.05; ***P <0.005.

A significant main effect of treatment (*P* = 0.019) was observed for picture recognition accuracy, whereby accuracy was significantly improved following NUTS as compared with PLA (see **Figure 3**A). This was driven by greater accuracy in rejection of novel (decoy) pictures (*P* = 0.025) following NUTS.

#### Picture recognition reaction time

A significant main effect of treatment (*P* = 0.004) was observed for picture recognition reaction time, whereby reaction time was significantly faster following NUTS as compared with PLA (see Figure 3B). This was driven by faster responses in correctly rejecting decoy pictures (*P* = 0.018).

#### Peg and ball errors

A significant treatment × visit interaction (*P* = 0.007) was observed for peg and ball errors. Pairwise comparisons revealed that significantly fewer errors were made on the peg and ball task following NUTS as compared with PLA during phase 2 (*P* = 0.044); and less errors were made when NUTS were consumed during phase 2 compared with phase 1 (0.026).

#### Rapid visual information processing accuracy

A significant treatment × visit interaction (*P* = 0.027) was observed for rapid visual information processing (RVIP) accuracy. Pairwise comparisons revealed significantly greater accuracy when NUTS were consumed during phase 1 as compared with phase 2 (*P* = 0.038).

#### Numeric working memory reaction time

A significant treatment × visit interaction (*P* = 0.018) was observed for numeric working memory reaction time. Pairwise comparisons revealed faster responses when PLA was consumed during phase 2 as compared with phase 1 (*P* = 0.038).

See supplementary tables for cognition (1) and mood (2) data.

### Metabolomics

Initially all samples underwent Principal Component Analysis (PCA) to identify sample outliers. From PCA, 4 samples were removed from the data set (data not shown). Next, a supervised PLSDA model was established to determine the extent of discrimination between the sample groups (**Supplementary Figure 1A**). Data from Supplementary Figure 1A shows that the treatment groups do not discriminate against each other implying only minor changes to the metabolome, and considerable similarity between groups exists. PC1 accounts for ∼20% variation and PC2 only ∼3%. Model parameters show low scores for data fit and predictive ability; (R2 < 0.5, Q2 < 0.5). Although changes to the overall metabolome were only minor, the overall patterns of changes are observable in a heat map of the top 100 features based on VIP score (Variable Importance in Projection) (**Supplementary Figure 1B**). As no significant changes to the metabolome following treatment were found, no attempt was made to identify discriminatory species through further analysis.

### Bacterial community

A total of 12,546,211 reads were screened for nonbacterial sequences and reduced to a total of 11,876,729 reads containing 5001 bacterial ASVs. Samples were then screened for contaminants based on the prevalence of bacteria in DNA extraction and sequencing negative control samples using the decontam package (35), further reducing the total library size to 10,894,606 and total number of ASVs to 4855. Counts of bacterial taxa within samples were then normalized by conversion to relative abundance to account for variation in sequencing depth.

**FIGURE 4:**
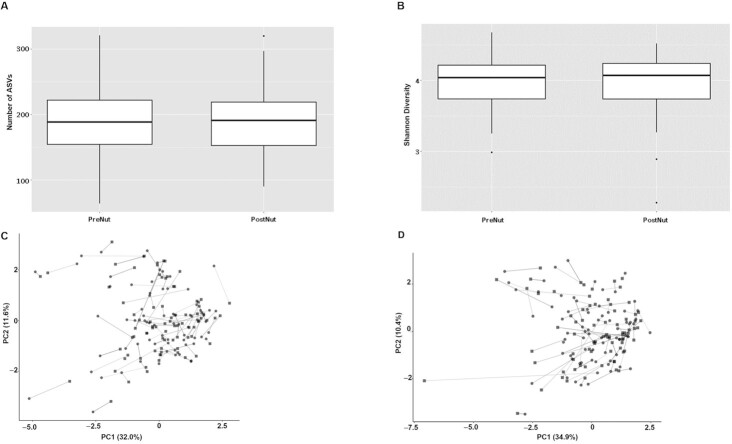
Comparison of the number of observed ASVs (Panel A) and Shannon Diversity (Panel B) in stool samples from healthy nonelderly adults between pre- and postmixed nut consumption for 4 wk. Principle co-ordinate analysis with Bray–Curtis dissimilarity between pre (circle) and post (square) intervention samples in healthy nonelderly adults receiving placebo (Panel C) and nuts (Panel D), each for 4 wk. ASV, amplicon sequence variant.

**FIGURE 5:**
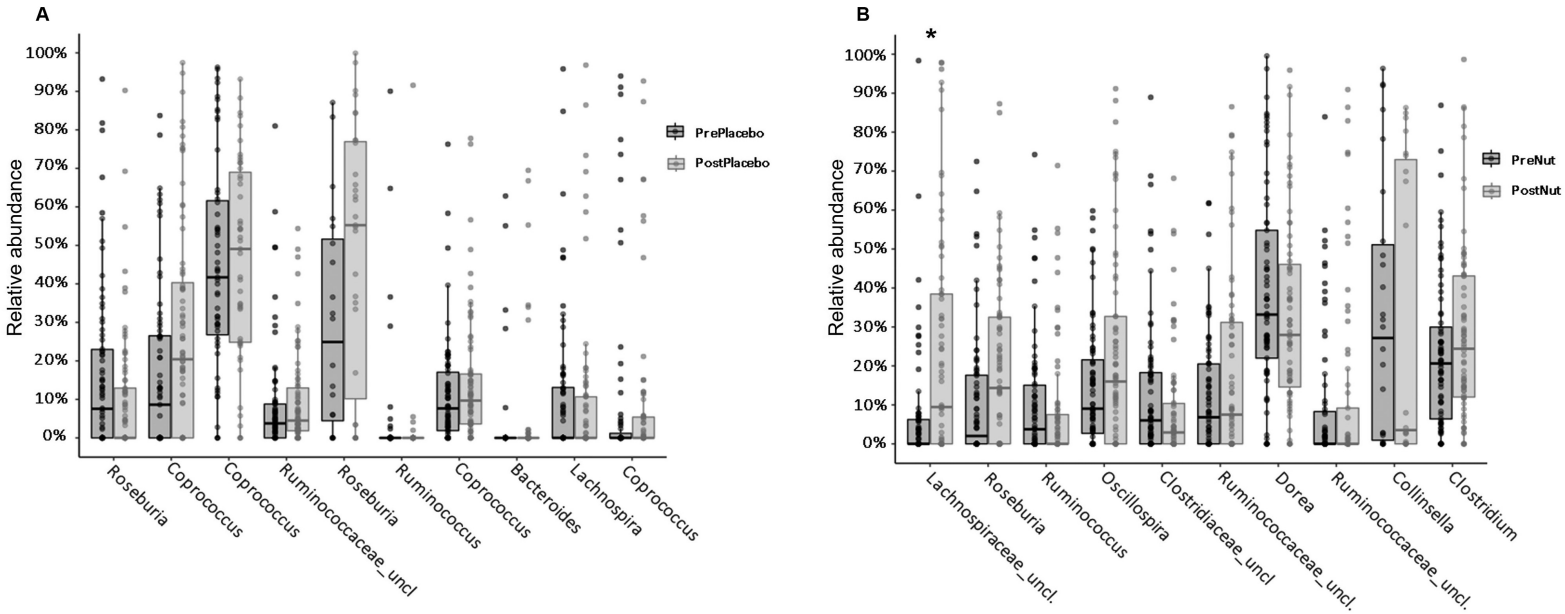
Comparison of top 10 ASVs showing the greatest difference between groups in the analyses. Pre- and posttreatment data in nonelderly adults who consumed placebo for 4 wk is shown. Wilcoxon analysis revealed no statistical significance between the relative abundance of these populations (Panel A). Comparison of top 10 ASVs showing the greatest difference between groups in the analyses. Pre- and posttreatment data in nonelderly adults who consumed mixed tree nuts for 4 wk. Wilcoxon analysis, False Discovery Rate (FDR) adj. revealed a statistically significance increase in the relative abundance of unclassified *Lachnospiraceae* (adj.*P* = 0.015) (Panel B). ASV, amplicon sequence variant.

There was no difference in the observed number of ASVs (*P* = 0.95) or the Shannon Diversity (*P* = 0.66) within the gut when the participants were supplemented with the intervention (**Figures 4**A and B). Adonis PERMANOVA showed that there was a significant dissimilarity between participants (*P* = 0.001) but not between pre- and postintervention samples (Figures 4C and D). There were no significant differences observed between pre- and postintervention samples for the placebo group (**Figure 5**A) but a significant increase in an unclassified *Lachnospiraceae* ASV was observed after intervention in the treatment group (*P* = 0.015) (Figure 5B). Additional analysis confirmed a significant main effect of treatment (*P* <0.001) whereby the unclassified *Lachnospiraceae* ASV was significantly increased following NUTS as compared with PLA, whilst controlling for baseline levels.

### Correlation

There was no significant correlation between change in the unclassified *Lachnospiraceae* ASV and change in picture recognition accuracy nor change in picture reaction time, whilst controlling for age, sex, years in education, and BMI.

## Discussion

Nut consumption led to improved picture recognition in terms of increased accuracy and faster reaction time in this sample of healthy nonelderly adults. Metabolomic analysis indicated only minor changes to the urinary metabolome following nut consumption. In terms of bacterial communities, an unclassified *Lachnospiraceae* was statistically enriched when participants were supplemented with nuts. Previous intervention studies have shown the effects of nuts on these parameters separately, but this is the first demonstration of the effects on cognition and gut microbiota within the same study.

The finding of improved picture recognition adds to a growing body of literature showing positive effects of nuts on cognition. Previous studies have largely focused on adults aged 50+ y and shown positive effects in those with mild cognitive impairment following Brazil nut (36), in healthy overweight following peanut (37), and in those at high risk of CVD following walnut (10) and Mediterranean diet plus mixed tree nuts (8, 38). Peanut supplementation and Mediterranean diet plus nuts have shown a benefit to memory (8, 37), whereas walnut supplementation had no effect on memory in a study of healthy young adults (11). This null finding may relate to an inability of the neuropsychological task employed to detect small changes in memory, particularly in a crossover design, due to a lack of parallel forms of the task. Moreover, previous evidence suggests that nut intervention studies targeting populations at higher risk of cognitive decline may find more favorable outcomes (39).

Nut consumption for 4 wk increased the abundance of an ASV belonging to the *Lachnospiraceae* family, members of which are among the main producers of SCFAs (40). This finding is consistent with a recent meta-analysis showing increased *Lachnospira* genera following nut consumption (41). Increases in *Roseburia, Clostridium*, and *Dialister*have also been shown following walnut (13) and almond (16) consumption, but these increases are not consistently observed (14, 15). Similarly, previously shown increases in α (15) and β (13–15) diversity were not evident here, which is consistent with a recent meta-analysis (41). The limited modulation of gut microbiota observed in the current study is not unexpected given the short and limited change to diet assessed; long-term (42) and/or large (43) shifts in dietary patterns may be needed to see profound changes in bacterial composition of the gut that overcome interindividual variability. Future work should extend bacterial community analysis to be totally untargeted deep metagenomic shotgun sequencing to determine whether there is a change at the strain level or a shift in the functional potential within the gut that cannot be detected by 16S rRNA sequencing.

Importantly, no association between changes in picture recognition outcomes and changes in *Lachnospiraceae* were observed in the current study following nut supplementation. Previous studies have shown cross-sectional associations between the gut microbiota and cognition or dementia risk but these findings and the direction of associations are not always consistent (44–47). One previous human study has investigated the relation between diet, cognition, and the gut microbiota. Adherence to a 12-mo Mediterranean diet intervention (NU-Age diet) in older adults resulted in higher relative abundances of a number of species, including *Faecalibacterium prausnitzii, Anaerostipes*, and *Roseburia*, which were weakly but positively associated with cognitive function (48). However, a subsequent analysis exploring the impact of the inflammatory index of the diet failed to show association between cognitive function and gut microbiota composition or diversity (49). One factor with the potential to impact cognition as well as the gut is blood supply. A 12-wk peanut intervention was shown to significantly increase small artery elasticity as well as cerebrovascular reactivity (CVR) in the left and right middle cerebral artery (MCA). Interestingly, increases in CVR in the left MCA were positively correlated with delayed memory and recognition (37), which may relate to the greater involvement of the left hippocampus in context-dependent episodic memory (50). A specific enhancement in ability to accurately reject novel pictures and to do so more quickly in the current study, may relate to the role of the hippocampus in pattern separation (51) and specifically, the left dentate gyrus/CA3, the volume of which has been shown to predict memory performance (52). Following walnut, amelioration of cognitive dysfunction in D-galactose model rats accompanied by the upregulation of hippocampal neurogenesis and the expression ofphosphorylated cyclic-AMP response element-binding protein (pCREB) and brain-derived neurotrophic factor (BDNF) in the hippocampus provides further support for hippocampal involvement in the effect of nuts on cognition (53).

Supplementation with mixed nuts failed to evince effects on mood in the current study. A previous study showed no effects of walnut on mood (11) but a follow-up analysis indicated improved mood in males only (54). Sex differences in response should be considered in future studies, particularly given the well-established differences in mood between men and women and previous demonstrations of sex-specific differences in the relation between mental wellbeing and dietary patterns (55). The sample included in the current study self-reported as healthy and experienced regular bowel movements; given the proposed relation between the gut and mood, effects may be more pronounced in those suffering gut dysbiosis or in those with disturbed mood and a stronger relation between the gut and brain may be evident in clinical populations (56).

Overall, the observed changes to the urinary metabolome were minor and supervised models were poor although some differences did exist. As the top identification matches did not relate to metabolic pathways perturbed by the intervention, these metabolomic changes were not investigated further. None of the discriminatory metabolites that were significantly increased in intensity had the same mass as that for known biomarkers of nut intake (for example, M-H m/z 227 for urolithin a, and urolithins were not measured in the current study). Urolithins appear to be the major urinary biomarkers of nut consumption (57) and these have been inversely linked to metabolic syndrome severity (58). Interestingly, the urolithin metabotype is thought to explain part of the interindividual variation in polyphenol metabolism (59) and can modulate the gut microbiome (60).

The current study explored the effects of nuts in addition to the diet rather than comparing to “unhealthy snacks”. Potential increases in body weight associated with this approach were not observed and meta-analysis indicates no increase in body weight, BMI, or waist circumference following nut consumption (61). Use of a crossover design overcomes issues of individual variation between participants and the 4-wk wash out included was deemed to be sufficient based on the lack of significant difference in microbial communities and the urinary metabolome across preintervention assessments. Interaction effects on cognition were observed. Numeric working memory showed beneficial effects for placebo when consumed during phase 2 compared with phase 1, which may be indicative of enduring effects of nuts from phase 1. However, effects on peg and ball and RVIP indicated differing effects of nuts depending on whether consumed in phase 1 or 2 and did not support the enduring effects of nuts. The issue of blinding a whole food was partially addressed by informing participants that the aim of the study was to explore effects of components of nuts and providing placebo capsules alongside nuts to facilitate this deception. Treatment guess data indicated that only 56% correctly guessed that they had received placebo during the final phase of the study.

In summary, the consumption of mixed tree nuts for 4 wk led to improvements on a picture recognition task in healthy nonelderly adults. This was accompanied by enrichment of an unclassified *Lachnospiraceae* ASV but limited changes to the urinary metabolome. To our knowledge this is the first study to show effects of a whole food on cognition and gut bacteria. Whilst these effects are limited in terms of both cognition and gut microbiota it is important to note the healthy and nonelderly status of the sample. More profound effects may be shown with higher quantities of nuts or in those at risk, such as those experiencing cognitive decline or in those suffering gut dysbiosis.

## Supplementary Material

nxac228_Supplemental_File

## Data Availability

Data described in the manuscript will be made available upon request.

## Notes

This study was supported by funding from the INC (International Nut and Dried Fruit Council).

The study was registered at clinicaltrials.gov with the identifier NCT03500601.

Author disclosures: The authors report no conflicts of interest.

Supplementary Tables 1 and 2 and Supplementary Figure 1 are available from the “Supplementary data” link in the online posting of the article and from the same link in the online table of contents at https://academic.oup.com/jn/.

Abbreviations used: ASV, amplicon sequence variant; COMPASS, computerized mental performance assessment system; CVR, cerebrovascular reactivity; HESI, heated spray ionization source; MCA, middle cerebral artery; PCA, Principal Component Analysis; PLA, placebo; PLSDA, Partial Least Squares Discriminant Analysis; RVIP, rapid visual information processing; VIP, Variable Importance in Projection.
