# Supplementary material for: Mixed Tree Nuts, Cognition, and Gut Microbiota: A 4-Week, Placebo-Controlled, Randomized Crossover Trial in Healthy Nonelderly Adults
Source: J Nutr. 2022 Oct 6;152(12):2778–88. doi: 10.1093/jn/nxac228 (PMC9840001; doi:10.1093/jn/nxac228)
Supplement: nxac228_Supplemental_File [file nxac228_supplemental_file.docx]

**Mixed tree nuts, cognition and gut microbiota: a 4-week, placebo-controlled, randomized crossover trial in healthy non-elderly adults – Haskell-Ramsay**

Supplementary table 1: Unadjusted means ± SD for cognitive task data for healthy non-elderly adults pre- and post-placebo for 4 weeks, and pre- and post-nut for 4 weeks

|  | | | **Baseline** | | **Day 28** | |  |  |
| --- | --- | --- | --- | --- | --- | --- | --- | --- |
|  | | N | Mean | SD | Mean | SD | p |  |
| Immediate Word Recall Correct (number) | Placebo | 79 | 7.23 | 2.12 | 7.59 | 2.18 | >0.1 | T |
|  | Nut | 79 | 7.38 | 2.52 | 7.36 | 2.29 | >0.1 | T x V |
| Immediate Word Recall Errors (number) | Placebo | 79 | 0.57 | 1.00 | 0.49 | 0.77 | >0.1 | T |
|  | Nut | 79 | 0.47 | 0.80 | 0.55 | 0.79 | >0.1 | T x V |
| Computerized Location Learning Total Displacement Score | Placebo | 77 | 10.79 | 9.40 | 9.55 | 8.93 | 0.081 | T |
|  | Nut | 77 | 10.78 | 10.55 | 11.95 | 12.16 | >0.1 | T x V |
| Computerized Location Learning Index | Placebo | 77 | 0.84 | 0.21 | 0.90 | 0.16 | 0.052 | T |
|  | Nut | 77 | 0.90 | 0.16 | 0.86 | 0.21 | >0.1 | T x V |
| Choice Reaction Time Accuracy (%) | Placebo | 79 | 97.90 | 2.12 | 97.77 | 2.04 | 0.094 | T |
|  | Nut | 79 | 97.90 | 2.31 | 98.13 | 1.90 | >0.1 | T x V |
| Choice Reaction Time (ms) | Placebo | 79 | 408.25 | 51.79 | 404.36 | 50.66 | >0.1 | T |
|  | Nut | 79 | 407.79 | 68.14 | 402.77 | 55.07 | >0.1 | T x V |
| RVIP Accuracy (%) | Placebo | 78 | 66.31 | 20.58 | 67.56 | 21.75 | >0.1 | T |
|  | Nut | 77 | 67.01 | 19.74 | 67.79 | 21.16 | 0.027 | T x V |
| RVIP RT (ms) | Placebo | 78 | 484.01 | 48.93 | 480.61 | 45.08 | >0.1 | T |
|  | Nut | 77 | 481.43 | 51.30 | 477.84 | 48.16 | >0.1 | T x V |
| RVIP False Alarms (number) | Placebo | 78 | 1.88 | 2.10 | 1.78 | 1.72 | >0.1 | T |
|  | Nut | 77 | 1.73 | 2.10 | 1.74 | 1.94 | >0.1 | T x V |
| Numeric Working Accuracy (%) | Placebo | 78 | 96.18 | 4.35 | 96.63 | 3.18 | >0.1 | T |
|  | Nut | 79 | 96.68 | 2.83 | 96.29 | 2.98 | >0.1 | T x V |
| Numeric Working Memory RT (ms) | Placebo | 78 | 770.72 | 145.54 | 759.73 | 135.49 | >0.1 | T |
|  | Nut | 79 | 771.87 | 152.32 | 762.98 | 161.32 | 0.018 | T x V |
| Logical Reasoning Accuracy (%) | Placebo | 75 | 85.47 | 14.87 | 86.86 | 14.93 | >0.1 | T |
|  | Nut | 78 | 84.55 | 15.84 | 86.92 | 14.40 | 0.063 | T x V |
| Logical Reasoning RT (ms) | Placebo | 75 | 3880.40 | 1376.24 | 3866.63 | 1361.62 | 0.094 | T |
|  | Nut | 78 | 4030.37 | 1332.91 | 3817.04 | 1271.43 | >0.1 | T x V |
| Stroop Accuracy (%) | Placebo | 77 | 97.40 | 2.68 | 96.97 | 2.77 | >0.1 | T |
|  | Nut | 76 | 97.21 | 3.19 | 97.26 | 2.44 | >0.1 | T x V |
| Stroop RT (ms) | Placebo | 77 | 676.64 | 108.61 | 667.51 | 98.95 | >0.1 | T |
|  | Nut | 76 | 669.90 | 92.94 | 675.00 | 101.38 | >0.1 | T x V |
| Stroop Interference RT (ms) | Placebo | 77 | 43.00 | 70.35 | 44.92 | 65.60 | >0.1 | T |
|  | Nut | 76 | 49.25 | 64.99 | 46.88 | 50.74 | >0.1 | T x V |
| Peg And Ball Thinking RT (ms) | Placebo | 77 | 2686.36 | 1399.67 | 2531.92 | 1267.15 | >0.1 | T |
|  | Nut | 77 | 2655.45 | 1330.46 | 2475.68 | 1228.89 | >0.1 | T x V |
| Peg And Ball Completion RT (ms) | Placebo | 77 | 8087.95 | 2012.37 | 7910.58 | 1750.73 | >0.1 | T |
|  | Nut | 77 | 8201.88 | 1672.44 | 7850.92 | 1709.71 | >0.1 | T x V |
| Peg And Ball Errors (number) | Placebo | 77 | 2.42 | 2.11 | 3.05 | 3.33 | >0.1 | T |
|  | Nut | 77 | 3.35 | 3.57 | 3.04 | 3.91 | 0.007 | T x V |
| Delayed Word Recall Correct (number) | Placebo | 78 | 5.49 | 2.17 | 5.97 | 2.31 | >0.1 | T |
|  | Nut | 79 | 5.38 | 2.43 | 5.63 | 2.47 | >0.1 | T x V |
| Delayed Word Recall Errors (number) | Placebo | 78 | 0.67 | 0.92 | 0.68 | 0.94 | >0.1 | T |
|  | Nut | 79 | 0.68 | 0.99 | 0.66 | 0.93 | >0.1 | T x V |
| Picture Recognition Accuracy (%) | Placebo | 78 | 93.76 | 7.49 | 92.51 | 8.43 | 0.019 | T |
|  | Nut | 78 | 92.61 | 7.10 | 94.19 | 6.23 | >0.1 | T x V |
| Picture Recognition RT (ms) | Placebo | 78 | 758.34 | 104.06 | 781.07 | 108.08 | 0.004 | T |
|  | Nut | 78 | 782.40 | 112.95 | 764.13 | 103.85 | >0.1 | T x V |
| Word Recognition Accuracy (%) | Placebo | 78 | 81.15 | 9.77 | 81.97 | 10.35 | >0.1 | T |
|  | Nut | 78 | 80.38 | 9.35 | 80.43 | 10.38 | 0.068 | T x V |
| Word Recognition RT (ms) | Placebo | 78 | 829.25 | 159.00 | 817.22 | 157.67 | >0.1 | T |
|  | Nut | 78 | 821.50 | 148.28 | 798.99 | 130.65 | >0.1 | T x V |
| Location Recall | Placebo | 77 | 0.60 | 1.39 | 0.34 | 0.98 | >0.1 | T |
|  | Nut | 77 | 0.81 | 2.06 | 0.22 | 0.84 | >0.1 | T x V |

ms=milliseconds; N=number of participants; RT=reaction time; RVIP=rapid visual information processing; SD=standard deviation; T=treatment effect; T x V=treatment x visit interaction

Supplementary table 2: Unadjusted means ± SD for Bond-Lader mood scales and Profile of Mood States (POMS) data for healthy non-elderly adults pre- and post-placebo for 4 weeks, and pre- and post-nut for 4 weeks

|  | | | **Baseline** | | **Day 28** | |  |  |
| --- | --- | --- | --- | --- | --- | --- | --- | --- |
| Bond-Lader | | N | Mean | SD | Mean | SD | p |  |
| Alert | Placebo | 79 | 61.40 | 14.46 | 63.33 | 14.95 | >0.1 | T |
|  | Nut | 79 | 62.84 | 13.95 | 63.85 | 13.35 | >0.1 | T x V |
| Calm | Placebo | 79 | 61.98 | 13.35 | 61.03 | 12.20 | >0.1 | T |
|  | Nut | 79 | 61.36 | 11.93 | 58.92 | 12.72 | >0.1 | T x V |
| Content | Placebo | 79 | 65.64 | 15.43 | 67.50 | 14.03 | >0.1 | T |
|  | Nut | 79 | 68.18 | 11.85 | 66.74 | 14.60 | >0.1 | T x V |
| POMS | | | | | | |  |  |
| Tension-Anxiety | Placebo | 78 | 6.25 | 4.31 | 6.12 | 4.34 | >0.1 | T |
|  | Nut | 79 | 6.11 | 3.89 | 6.01 | 4.70 | >0.1 | T x V |
| Depression-Dejection | Placebo | 78 | 3.22 | 5.81 | 3.06 | 4.70 | >0.1 | T |
|  | Nut | 79 | 2.29 | 3.45 | 3.19 | 4.89 | >0.1 | T x V |
| Anger-Hostility | Placebo | 78 | 2.85 | 4.30 | 2.36 | 3.35 | >0.1 | T |
|  | Nut | 79 | 2.51 | 3.76 | 2.78 | 4.71 | >0.1 | T x V |
| Vigour-Activity | Placebo | 78 | 16.04 | 6.51 | 16.07 | 6.70 | >0.1 | T |
|  | Nut | 79 | 15.78 | 5.93 | 16.40 | 6.25 | >0.1 | T x V |
| Fatigue-Inertia | Placebo | 78 | 4.94 | 4.14 | 4.52 | 3.94 | >0.1 | T |
|  | Nut | 79 | 4.19 | 3.75 | 4.47 | 4.00 | >0.1 | T x V |
| Confusion-Bewilderment | Placebo | 78 | 7.77 | 4.50 | 7.52 | 4.19 | >0.1 | T |
|  | Nut | 79 | 7.42 | 4.21 | 7.08 | 4.07 | >0.1 | T x V |
| Total Mood Disturbance | Placebo | 78 | 8.96 | 20.67 | 7.51 | 19.21 | >0.1 | T |
|  | Nut | 79 | 6.74 | 16.00 | 7.13 | 21.67 | >0.1 | T x V |

N=number of participants; SD=standard deviation; T=treatment effect; T x V=treatment x visit interaction


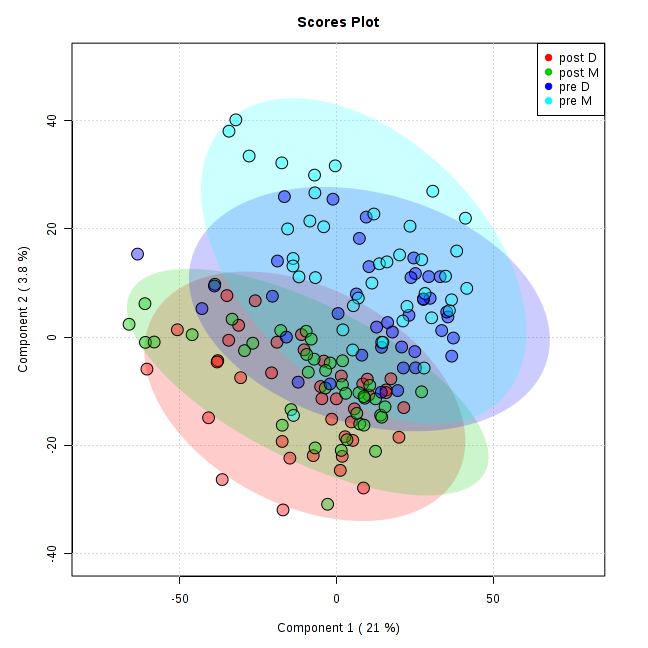

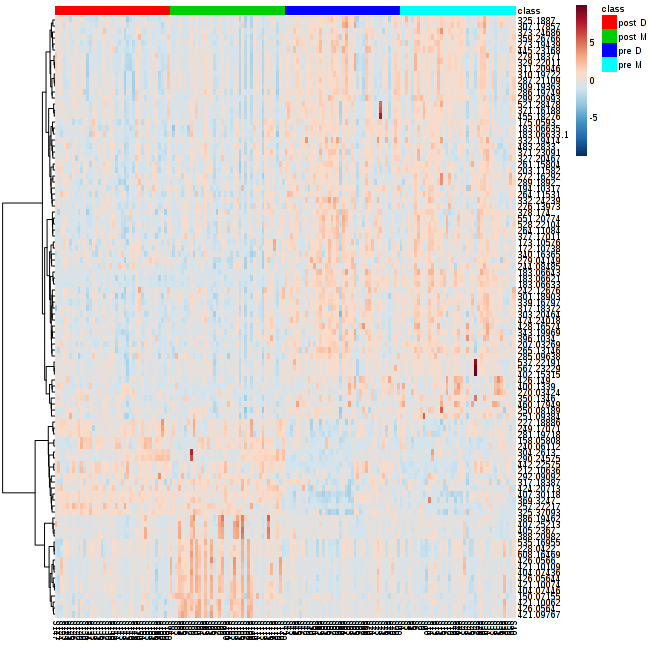


Pre-Nut

Post-Nut

Pre-Placebo

Post-Placebo

Supplementary figure 1 - Partial Least Squares Discriminatory Analysis (PLSDA) of the urinary metabolome pre- and post-placebo for 4 weeks, and pre-and post-nut for 4 weeks in healthy non-elderly adults

A

B

A score plot is shown in (A) and highlights the overlap between the groups such that it was not possible to discriminate post treatment. Nevertheless a heat map of the top 100 ion features (B) does show some ions that are either increased or decreased following the nut treatment (green).
